# Supplementary material for: Krebs Cycle Intermediate-Modified Carbonate Apatite Nanoparticles Drastically Reduce Mouse Tumor Burden and Toxicity by Restricting Broad Tissue Distribution of Anticancer Drugs
Source: Cancers (Basel). 2020 Jan 9;12(1):161. doi: 10.3390/cancers12010161 (PMC7017074; doi:10.3390/cancers12010161)
Supplement: Supplementary file 1 [file cancers-12-00161-s001.pdf]

Supplementary Table 1. Organ distribution of free DOX, DOX-loaded CA NPs, DOX-loaded CMCA NPs, and DOX-loaded α-KAMCA NPs at 2 h of treatment (*n* = 5).

Organs	Free DOX (%)	DOX–CA (%)	DOX–CMCA (%)	DOX–α-KAMCA (%)
**Liver**	0.35	1.07	0.35	0.96
**Spleen**	0.08	0.14	0.08	0.14
**Heart**	0.41	0.2	0.17	0.11
**Lungs**	0.08	0.08	0.08	0.08
**Brain**	0.13	0.42	0.28	0.25
**Kidney**	0.42	0.2	0.37	0.22
**Tumor**	0.08	0.37	0.45	0.62

Supplementary Table 2. Organ distribution of free DOX, DOX-loaded CA NPs, DOX-loaded CMCA NPs, and DOX-loaded α-KAMCA NPs at 24 h of treatment (*n* = 5).

Organs	Free DOX (%)	DOX–CA (%)	DOX–CMCA (%)	DOX–α-KAMCA (%)
**Liver**	1.38	1.19	0.35	0.36
**Spleen**	0.08	0.14	0.09	0.08
**Heart**	0.62	0.4	0.17	0.11
**Lungs**	0.08	0.08	0.08	0.08
**Brain**	0.47	0.6	0.4	0.25
**Kidney**	0.42	0.32	0.37	0.16
**Tumor**	0.08	0.23	0.3	0.4

Supplementary Table 3. Estimation of serum drug concentration following an intravenous injection of free DOX, DOX–CA NPs, DOX–CMCA NPs, and DOX–α-KAMCA NPs (n = 5).

Formulations	2 h (%)	24 h (%)
**Free DOX**	2.02	1.71
**DOX–CA**	9.83	5.93
**DOX–CMCA**	6.19	4.33
**DOX–α-KAMCA**	16.02	8.04

Supplementary Table 4. Weight of different organs of mice from tumor-bearing untreated mice group, only DOX-treated group, and DOX-loaded NP-treated group (n = 5).

Organs	Tumor-bearing untreated group (mg)	DOX-treated group (mg)	DOX-loaded NP-treated group (mg)
**Liver**	1102	1102	1102
**Spleen**	509	509	509
**Heart**	116	116	116
**Lungs**	187	187	187
**Brain**	355	355	355
**Kidney**	230	230	230
**Tumor**	181	181	181
